# Clinical value of new drugs in acute myeloid leukemia

**DOI:** 10.1097/HS9.0000000000000223

**Published:** 2019-06-30

**Authors:** Gert Ossenkoppele

**Affiliations:** Amsterdam UMC, Location VUMC, The Netherlands

**Take home messages**

- There is an urgent medical need for improving treatment of AML.
- Emerging targeted therapies for patients with AML are successfully entering the clinic.
- Many immunotherapeutic approaches in the treatment of AML are currently explored.

## Introduction

Acute myeloid leukemia (AML) is a clonal neoplasm derived from myeloid progenitor cells with a varying outcome depending on risk profile mainly defined by cytogenetic and mutational abnormalities. The initial treatment goal is the achievement of complete remission (CR) defined already for over 40 years by morphology. Preferably this should be CR without measurable residual disease (MRD) as it is now defined in the ELN 2017 recommendation.^1▪^ Without additional post-remission treatment, however and despite high CR rates are achieved, the majority of patients relapse.

## State of the art and future perspective

For over 50 years the standard of care (SOC) consisted of a combination of an anthracycline and cytosine-arabinoside (7 + 3) for those patients that can be treated intensively.^2^ SOC for unfit patients can be hypomethylating treatment or low dose ARA-C. The progress that has been made in the last 40 years in the outcome of AML is mainly due to better supportive care and the increased application of as well as better selection of patients suitable for allogeneic stem cell transplantation (alloSCT). In many cases of AML, alloSCT offers the best prospects of cure. Indeed, AML is the most frequent indication for alloSCT as indicated by data from both the EBMT and IBMTR and the numbers are growing year by year.^3^ Because addition of classical cytotoxic drugs to SOC has not resulted in a better outcome it is obvious that new treatment modalities are necessary for further improvement. Application of NGS has unraveled the mutational landscape of AML instrumental to serve as a refinement of prognostic classification but also as a toolbox for new targets for drugs.^4▪^

Many new drugs are currently in development, quite a number have been approved by the EMA and/or FDA in the past 2 years (see Table ^1^).^5^

– Vyxeos (CPX-351) is a liposomal formulation of cytosine-arabinoside and daunorubicin at a 5:1 molar ratio with a preferential uptake in leukemic blasts. It has been shown to significantly improve median overall survival vs 7 + 3 (9.56 vs 5.95 months; HR 0.69;. Although a slightly longer hematological recovery was observed early mortality rates were lower for CPX-351 as compared to 7 + 3 (5.9% and 10.6%, respectively) through day 30 and day 60, (13.7% and 21.2%, respectively).^6^
– *FLT3* mutations and *IDH* mutations are examples that have led to the development of specific inhibitors. Midostaurin is the first *FLT3* inhibitor that in addition to intensive chemotherapy has shown of benefit in *FLT3*mutated AML.^7▪^ Overall survival was significantly longer in the midostaurin group than in the placebo group (hazard ratio for death, 0.78) as was event-free survival (hazard ratio for event or death, 0.78). However, midostaurin is not a specific inhibitor, therefore, quite a number of more selective inhibitors of FLT3m are currently under investigation and are very promising in Phase I/II settings. Gilteritinib approved by FDA on 28th November 2018 and quizartinib are most advanced.
– *IDH* is a critical metabolic enzyme in the citric acid cycle. *IDH1* is located in cytoplasm and *IDH2* in mitochondria. *IDH1/2* mutation produces 2-hydroxyglutarate (2-HG) that acts as an oncogene and blocks normal cellular differentiation important in the pathophysiology of AML. Enasidenib and Ivosidenib are *IDH2* and *IDH1* mutation inhibitors, respectively, that as monotherapy have proven to be highly effective in refractory and relapsed AML. These drugs are now also under investigation in the upfront setting in fit and unfit elderly either as monotherapy or in combination with low intensity therapy.^8^–^10^ Enasidenib has been approved by FDA in August 2017 and has granted orphan drug designation by EMA.
– BCL-2 protein is another promising therapeutic target for AML. Overexpression enhances survival of AML cells and is associated with chemotherapy resistance in AML and poor survival.^11▪^ Venetoclax is a potent, orally available, selective BCL-2 inhibitor that in combination with low intensity treatment (HMAs or LDAC) shows very high CR rates, even MRD negativity is achieved, and durable responses.^12^,^13^ Venetoclax has been approved by FDA last November.
– Aberrant Hedgehog signaling is critical for leukemia stem-cell survival and expansion. Overexpression of Hedgehog pathway components has been shown in chemotherapy-resistant myeloid leukemia cells. Inhibition of this pathway enhanced the sensitivity to chemotherapy. The addition of glasdegib, a Hedgehog pathway inhibitor, to low dose ARA-C (LDAC) resulted in an improvement in OS compared with the standard therapy of LDAC. Glasdegib is approved in November 2018 in the USA for use in combination with low-dose cytarabine for the treatment of newly-diagnosed acute myeloid leukemia (AML) in patients aged ≥75 years or those who have comorbidities that preclude use of intensive induction chemotherapy.

**Table 1:**
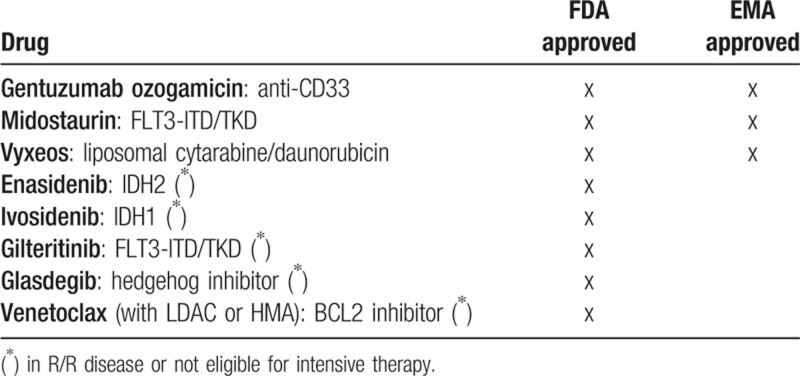
Recently Approved Drugs by EMA and/or FDA for AML

The list of new drugs currently investigated in AML is extended and although many are in early development the future looks bright for the patient with AML.^14^

Besides transplant-based immunotherapy other forms of immunotherapy are emerging.^14^,^15^

Leukemic cells express antigens that can be recognized by monoclonal antibodies: naked as well as conjugated antibodies (ADC) are used for targeted tumor eradication. Until now only gemtuzumab ozogamycin (mylotarg) has been proven to be successful especially in good risk AML.^16▪^ New ADCs targeting other surface molecules (CD123, CLL-1) are currently under investigation).

Bispecific antibodies targeting a leukemia specific antigen on the one end and a T cell recognizing antigen on the other end bring together T cells and tumor cells in order to eradicate the leukemia cells.^17^ In ALL this concept already has shown to be very successful. Blinatumumab, a CD19/CD3 BiTE antibody is approved for ALL. Antigens of interest for AML are CD33, CD123, and CLEC12 and bispecifics directed against these are now explored in Phase I studies.

Immune checkpoint inhibitors are in early stage of development in AML as is also the case for CART cell therapy and the application of AML dendritic cell vaccines.^18^–^20^ The translation of the success of CARTs in B-cell neoplasia to AML because AML-associated target antigens have a more ubiquitous expression pattern overlapping with healthy hematopoiesis.

Results of ongoing studies have to be awaited.

In summery many new drugs are emerging in AML. Many targeted agents are successfully entering the clinic. For immunotherapy apart from the alloSCT it is still early days and we have to await the outcomes of the multitude of studies that are ongoing.
